# Immune checkpoint inhibitors in neuroendocrine tumors: A single institution experience with review of literature

**DOI:** 10.18632/oncotarget.23753

**Published:** 2017-12-28

**Authors:** Aman Chauhan, Millicent Horn, Gray Magee, Kurt Hodges, Mark Evers, Susanne Arnold, Lowell Anthony

**Affiliations:** ^1^ Markey Cancer Center, University of Kentucky, Lexington, KY, USA; ^2^ School of Medicine, University of Kentucky, Lexington, KY, USA; ^3^ Department of Pathology, University of Kentucky, Lexington, KY, USA

**Keywords:** immune check point inhibitor, neuroendocrine tumors

## Abstract

This unique case series and review of literature suggests that immune checkpoint inhibitors may have clinical activity in neuroendocrine tumors.

**Objective:**

Summarize advances of immuno-oncology in neuroendocrine tumors with the help of a case series.

**Design:**

Case series and review of literature.

**Intervention or Exposure:**

The patients were treated with immune checkpoint inhibitors (pembrolizumab or nivolumab).

**Main Outcome(s) and Measures(s):**

Life expectancy, quality of life, disease progression.

**Results:**

Maximum durable response of 16 months in one of the patients so far. All patients showed improvement in quality of life before disease progression. Two out of four are still on therapy. None of the patients experienced immune checkpoint inhibitor associated side-effects. All patients had failed standard of care therapy prior to the initiation of immune checkpoint inhibitors and were on the verge of hospice.

**Conclusions:**

Immune checkpoint inhibitors have revolutionized cancer management and the last 5 years have seen a rapid expansion in the indications for this class of drug. Neuroendocrine tumors, unfortunately, have been slow to catch on to the immuno-oncology, partly due to difficulties in establishing relevant preclinical neuroendocrine tumors models for immune-oncology studies. In this manuscript, we review the current status of immunotherapy in neuroendocrine tumors.

## INTRODUCTION

Immune checkpoint inhibitors have rapidly advanced in medical oncology. Programmed death-ligand 1 (PD-L1) is a transmembrane protein that binds to the programmed death-1 receptor (PD-1) during immune system modulation. The PD-1 receptor is typically expressed on cytotoxic T-cells and other immune cells while PD-L1 ligand is typically expressed on normal cells [31]. Under normal conditions, cells use the PD-1/PD-L1 interaction as a mechanism of protection against immune recognition *via* inhibiting the action of T-cells thus downregulating the immune response such that inactive T-cells are exhausted, cease to divide and eventually die by programmed cell death or apoptosis [30]. Studies have shown that numerous types of tumor cells upregulate the expression of PD-L1 as a mechanism to evade the immune response [31]. Activated T-cells recognize the PD-L1 marker on the tumor cell (similar to that of a normal cell) and render the cytotoxic T-cell inactive and thus the tumor cell escapes the immune cycle for elimination and is able to proliferate [31]. Despite a tremendous thrust of anti PD-1 and PDL-1 agents, the realm of neuroendocrine tumors (NETs) seems to be relatively untouched. This manuscript summarizes current advances of immuno-oncology in NETs with the help of a case series (Table 1).

**Table 1:** Summary of immune checkpoint inhibitor experience in neuroendocrine tumors

Patient Age	Diagnosis	Grade	Treatment	Prior Treatments	Duration of Treatment	Outcome
43	Stage IV pNET, Non functional	II	Pembrolizumab	somatostatin analog, sunitinib, capecitabine and temozolamide, everolimus and fosbretabulin	16 months	Progressed after 16 months of stable disease; Initially KPS increased from 60% to 90%; 20 pound weight gain and enjoyed a good quality of life for over a year until progression.
49	Stage IV NET of unknown primary, Non functional	II	Nivolumab	somatostatin analogs, everolimus, capecitabine and temozolomide	6 months	KPS increased from 70% to 90% initially. Progressed after 6 months on treatment
71	Stage IV pNET, Gastrin producing	I	Nivolumab	somatostatin analog, everolimus, capecitabine and temozolomide, fosbretabulin	6 months	Stable disease per imaging
75	Stage IV, Bronchial NET, Non functional	I	Pembrolizumab	Somatostatin analog, everolimus, XRT	3 months	Stable disease per imaging

## CASE SERIES

### Case 1

**Figure 3:**
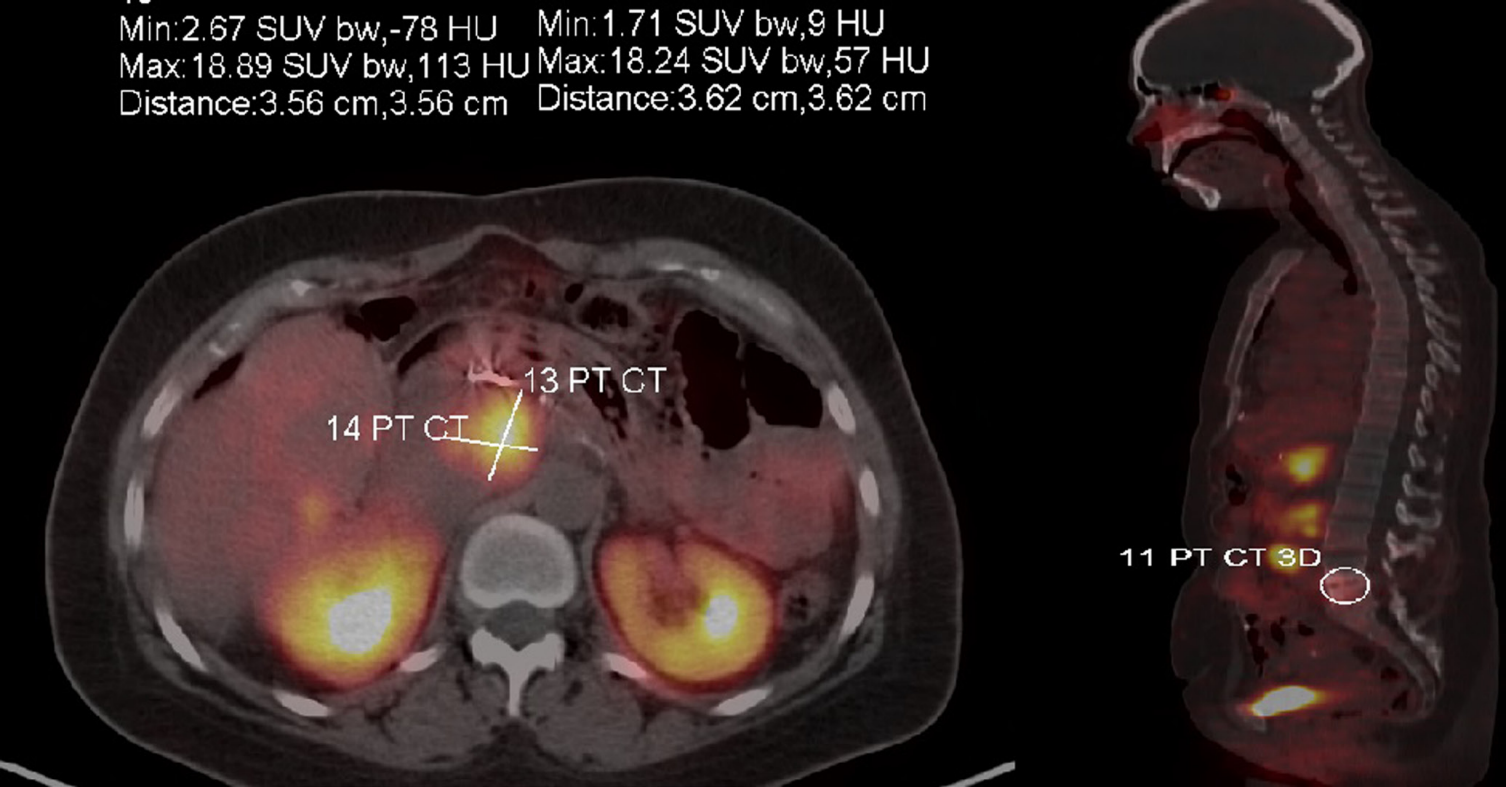
Gallium DOTATATE scan sowing disease burden at the time of progression on immune checkpoint inhibitor therapy (case 1)

A 43-year-old female was in her usual state of health until about January of 2011 when she developed nausea, vomiting and diarrhea. Diarrhea persisted for a couple of months and she sought medical help in March 2011. Initial conservative management followed by a detailed workup done by a gastroenterologist was negative. Later, in August 2011 the patient presented to ED with complaints of melena. Her CT scan revealed a 9-cm mass in the tail of her pancreas with hepatic metastasis. Liver biopsy confirmed grade 2 neuroendocrine tumor (Ki-67 of 6%). The patient was initiated on Sandostatin LAR 30 mg every 30 days. She noticed rapid improvement in her energy level. She subsequently underwent Y90 radio-embolism of hepatic metastatic disease first in the right lobe of the liver followed by the left lobe in the months of September and October 2011. The patient had a stable course until December 2011, she had another episode of GI bleed. In January 2012 she underwent partial pancreatectomy, splenectomy, partial gastrectomy and left hepatic lobectomy. She was started on sunitinib in February 2012, which she had to rapidly discontinue within a month due to severe fatigue. She was started on capecitabine and temozolomide (CAPTEM) in April 2012. The patient tolerated CAPTEM well and started gaining weight. An abdominal MRI from May 2013 showed mild worsening of some of the hepatic lesions, however the rest of the disease was stable. She underwent two more doses of Y-90 radioembolism. She was continued on long acting somatostatin analog and CAPTEM and her subsequent surveillance scan in November 2013 showed stable disease. A follow-up MRI of the abdomen in February 2014 showed mild progression of one of the hepatic lesions. She was taken off CAPTEM and started on everolimus 10 mg daily. We had to reduce the dose of everolimus to 7.5 mg daily due to stomatitis. The patient did exceptionally well on the reduced dose of everolimus and had stable disease until October 2015, at which time she was enrolled into a Phase I clinical trial of fosbretabulin for progressive disease in the liver and retroperitoneum. The patient got the first dose of fosbretabulin in November 2015. She only received three cycles of fosbretabulin before she developed disease progression in the left supraclavicular and left axillary lymph nodes. In February 2016 she was started on off-label pembrolizumab. She had stable disease on pembrolizumab for 16 months before her disease progressed in axilla and breast. Figure 3 shows the current disease burden of patient with help of gallium DOTATATE scan.

### Case 2

A 49-year-old woman was in her usual state of health until 2014 when she presented with complaints of a 6-month history of a non-productive cough, and was found to have pneumonia of the right lung. Scans revealed a right upper lobe lesion. A subsequent bronchoscopy revealed a right upper lobe tumor. On 8/19/2014, the patient underwent an upper lobectomy, and pathology confirmed the diagnosis of stage III a, typical carcinoid (well differentiated NET). Peri-bronchial lymph node (1 out of 7) was also positive for typical carcinoid. In May of 2015, the patient felt a nodule under her skin in her left upper back. A biopsy was consistent with a grade II well differentiated metastatic neuroendocrine tumor. Resection was performed with negative margins, and a postoperative CT was negative for additional metastases. Patient was surveilled with imaging every 3-4 months. An MRI in in fall of 2015 demonstrated progression of the disease in the liver and thorax. She was started on CAPTEM. Unfortunately, patient progressed after five months on CAPTEM. Patient was started on everolimus. She progressed after 3 months and was switched to daily sunitinib. A CT scan in fall of 2016 demonstrated progression of multiple hepatic lesions. She was started on off-label nivolumab (240 mg i.v. every 2 weeks). Patient had stable disease for 6 months on nivolumab before progression and as of now patient has opted hospice. It is to be noted that prior to stating nivolumab patient was losing weight and reported fatigue. Patient had significant improvement in her energy levels and gained 10-15 pounds during first 4-5 months on treatment.

### Case 3

A 71-year-old male underwent a distal pancreatectomy, splenectomy, and a partial gastric resection on 8/12/2012 under the suspicion of the presence of a pancreatic tumor. The resection revealed a grade II pancreatic NET with 5/18 positive nodes. There was evidence of lymphovascular and perineural invasion. In February of 2015, he began treatment on E2211 clinical trial comparing single agent temozolomide to temozolomide + capecitabine, and was randomized to receive temozolomide. After progression, he was switched to CAPTEM in May of 2015. CAPTEM was discontinued in September of 2015 due to progression. He was then switched to long acting somatostatin analog. A CT scan from 10/20/2015 demonstrated interval hepatic metastatic disease progression. He was then started on phase I clinical trial of fosbretabulin in December of 2015. Fosbretabulin is a vascular disrupting agent currently under clinical investigation. Unfortunately, CT scan on 5/23/2016 showed hepatic progression. He was switched to everolimus and progressed after 5 months of therapy. He began off-label nivolumab (240 mg i.v. every 2 weeks) in December of 2016. At six months, the patient continues to tolerate the treatment well and shows radiological stable disease.

### Case 4

A 75-year-old woman was initially found to have a left lower lung lobe (LUL) lesion during pre-operative evaluation for right total knee replacement in December 2014. CT demonstrated a 1.4cm LUL lesion which was biopsied at outside hospital. Biopsy consisted of 2 FNA which contained neuroendocrine cells positive for synaptophysin, chromogranin, CD56, and CK7 as well as TTF-1. Ki-67 staining was low. There was no evidence of increased mitosis and no necrosis. Morphology and immunohistochemistry were compatible with a well-differentiated neuroendocrine tumor, although the precise grade (typical *vs* atypical carcinoid) could not be definitively determined due to limited tissue. Octreotide scan done at outside hospital was noted to be positive with positivity in the left upper lobe and right mid/lower pulmonary hilum and focal lesion in the liver. She has no evidence of carcinoid syndrome. As it was felt that patient has at least a stage IIIB disease with biopsy proven positive LN in the contralateral mediastinum. She underwent a LUL video assisted thoracoscopy (VATS) wedge resection. VATS demonstrated a 1.5 cm moderately to poorly differentiated adenocarcinoma with invasion in the visceral pleura. Surgical margins were negative and tumor was positive for TTF-1. Lymph nodes were negative (pT2 N0 M0, stage IA). She subsequently underwent a bronchoscopy with an ultrasound-guided biopsy and mediastinoscopy. At this procedure, a left lower lobe primary carcinoid tumor of the lung was found, but not resected at that time since it would require a lobectomy. Pathology demonstrated this right lower lobe biopsy to show a well-differentiated neuroendocrine tumor. A level 7 lymph node on the right was also positive for neuroendocrine tumor, well-differentiated. Peritracheal nodes were negative.

In May 2016, she has developed a chronic cough. Subsequent workup demonstrated progressive disease in her chest. Interventional pulmonology and radiation oncology recommended SBRT for obstructive lung lesion. Post radiation, she was started on somatostatin analogs along with everolimus. Gallium DOTATATE PET confirmed progressive metastatic disease in thorax and abdomen. The patient was started on off-label pembrolizumab in June 2017 and her first three monthly surveillance CT scan showed stable disease.

## DISCUSSION

**Figure 1:**
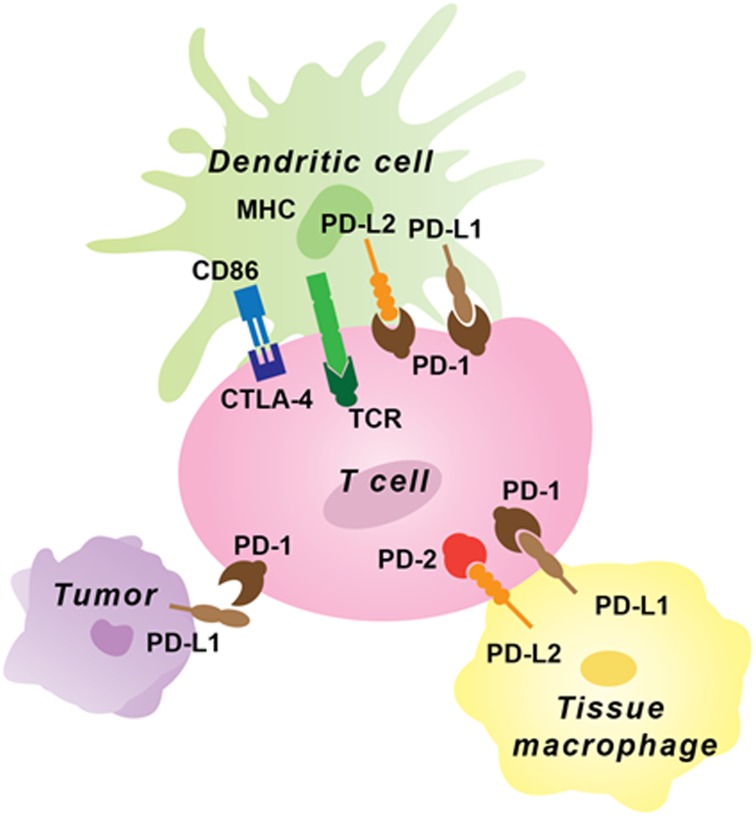
Immune checkpoint inhibitor expression on tumor and immune cells

One of the hallmarks of a healthy state is homeostasis between immune activation and degree of inflammatory response. Be it an infectious stimulus or tumors, a well-balanced interplay of immune activation and subsequent shutdown once the aberrant signal protein is eliminated is paramount. An exception to this leads to a pathological state. As scientists began to understand the biology of cancer, it became quite clear that one of the mechanisms by which cancer cells evade our immune response is with the help of immune checkpoints. As noted earlier, most of the known immune checkpoints are in a set of cellular receptors or ligands, which once activated blunt T cell response against cancer cells. Figure 1 shows the mechanism of action of common immune checkpoint proteins. This led to development of antibodies towards these immune checkpoints with a goal of helping the host immune system to re-recognize these tumor cells as foreign.

Traditionally the mutagenic burden of a tumor type has been thought to predict activity of immune oncology drugs [1]. This hypothesis stems from impressive responses noticed in high mutational burden tumors which may have more neoantigens like lung cancer and melanoma when they are treated with immune checkpoint inhibitors [2, 3].

**Figure 2:**
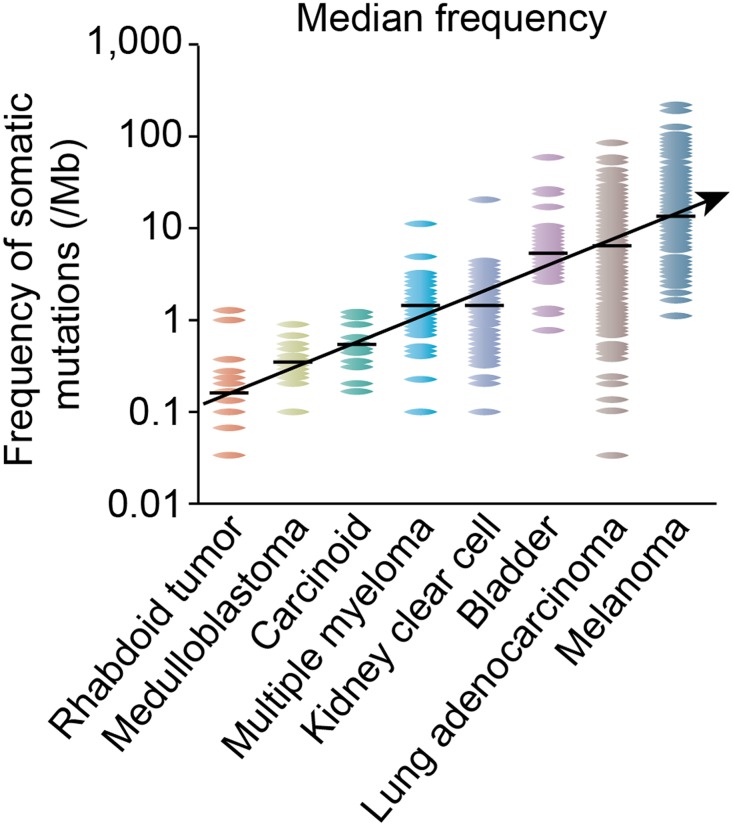
Somatic mutational burden in varying tumor types

This led to rapid advances in early phase clinical trials in many such tumor types. However, tumors with low mutational burden were left out without any real evidence of lack of PD-1/PD-L1 expression or efficacy. Figure 2 is adapted from a seminal paper from Nature Reviews and shows low mutational burden of well differentiated neuroendocrine tumors (carcinoid) [4].

The notion that low mutational burden predicts poor response to immune checkpoint inhibitors has come into question by some of the leading experts in immune-oncology. This could be due to three factors: Firstly, the data regarding PD-1/PD-L1 expression is expanding even in low mutational burden tumors [5, 24]. Secondly, there is growing evidence that PD-1/PDL-1 expression is not static and can change with tumor progression and based on tumor microenvironment [6]. Lastly, PD-1/PD-L1 expression can be augmented with help of immunomodulators. In fact, the newer immune checkpoint inhibitor clinical trials are utilizing concurrent cytotoxic T lymphocyte antigen 4 (CTLA-4) inhibitor, Indoleamine 2,3-dioxygenase inhibitors (IDO), interferon (INF), radiation and cytotoxic chemotherapy to enhance the efficacy of immune checkpoint inhibitors [7–10].

### Immuno-oncology in neuroendocrine tumors

Oberg et al. were among the first researchers to report the potential benefit of interferon (INF) in neuroendocrine tumors. They treated 9 NET patients, including six with carcinoid syndrome, with daily intramuscular INF for three months. All six syndromic patients experienced temporary relief in carcinoid syndrome symptoms and concomitant decline in urinary 5 HIAA levels [11].

Moertel et al. treated 27 of their neuroendocrine tumor patients with interferon. 24 out of 27 patients had carcinoid syndrome. 20% of the patients had an objective decrease in tumor size. 39% of functional NET patients experienced reduction in urinary 5 HIAA (>50%) levels. These effects were transient for about 4-7 weeks. Moreover, authors recommended against the use of INF due to toxic side-effects which included fever, fatigue, and weight loss [12].

In a subsequent prospective randomized clinical trial of octreotide (*n* = 35) *versus* octreotide with INF (*n* = 33) for metastatic midgut carcinoid tumor patients, a superior progression free survival was evident for the combination arm; however, no significant difference was seen in overall survival [13].

Another randomized clinical trial done at around same time compared the efficacy of lanreotide (*n* = 25) to INF-alpha (*n* = 27) and the combination of lanreotide and INF-alpha (*n* = 28) in metastatic gastroenteropancreatic neuroendocrine tumor patients. The study concluded that the experimental agents had comparable anti-neoplastic activity in NETs but response rates were very poor. Only 19 patients out of 80 showed stable disease at 12 months [14]. Arnold et al. prospectively studied interferon plus octreotide (*n* = 54) *versus* octreotide alone (*n* = 51) in 105 gastroenteropancreatic tumor patients. The median survival was 54 months in the combination arm *vs* 32 months in the octreotide only arm. The study could not establish superiority of the combination arm due to lack of statistical significance [15].

Based on the above mentioned weak evidence, marginal benefit and lack of therapeutic options, NCCN recommends (category 3) INF as one of the potential treatment choices for metastatic gastrointestinal and thoracic NET [16]. This recommendation fortunately has fallen off favor in general community practice.

### Modern immuno-oncology and NETs

Ipilumumab, a monoclonal antibody against cytotoxic T lymphocyte antigen 4 (CTLA4), was approved in March 2011 and was the first immune checkpoint inhibitor to show an overall survival advantage in metastatic melanoma [17]. Theoretically, the efficacy of immune checkpoint inhibitors should be affected by the presence of tumor infiltrating lymphocytes (TILs). Increased TILs should in principle enhance the activity of anti-CTLA4 drugs. Although we have no direct prospective data on the efficacy of CTLA-4 agents in NETs, there is indirect data to suggest that immune checkpoint inhibitors might have activity in NETs.

Ryschich et al. were the first to demonstrate the presence of CD3+ T cells in pancreatic neuroendocrine tumors (PNET) [18]. Later, a series published by Kat et al. from Memorial Sloan Kettering analyzed post-operative tumor specimens of 87 patients with primary PNET for TILs. They found a statistically significant difference in survival of intermediate grade (Ki 67 2-20%) PNET based on TIL density. Patients with dense T cell infiltration in tumor tissue had a median recurrence-free survival of 128 months as compared to 62 months in the subset with low TILs (*p* = 0.05) [19].

Drug development for high grade NETs usually follows trends seen in small cell lung cancer, a pulmonary high grade NET. Reck et al. were among the first to study ipilumumab in extensive stage small cell lung cancer (SCLC). Phased ipilumumab with carboplatin/paclitaxel *vs* carboplatin/paclitaxel alone showed a statistically significant difference in irPFS (HR (hazard ratio) = 0.64; p = 0.03). Although the improvement in irPFS did not translate into overall survival it was nevertheless indicative of drug efficacy in this tumor type [20].

Checkmate 032 evaluated nivolumab *vs* nivolumab plus ipilumumab in 128 SCLC patients who had progressed on prior platinum-based regimens. The overall response rate was 18% *vs* 32.6% for the combination arm [21]. The therapy showed significant antitumor activity, while maintaining durable responses and a manageable toxicity profile in patients with progressed SCLC. Keynote 028 evaluated pembrolizumab in PD-L1 positive progressive SCLC patients. Out of 24 treated patients one patient had complete response and 8 had partial response. ORR was 37.5% [28]. Whether these agents will prove to be effective in non-pulmonary high-grade NETs is yet to be seen.

A recent retrospective study from Korea evaluated PD-L1 expression in metastatic gastroenteropancreatic (GEPNET) patients through immunohistochemical analysis. 7 out of 32 patient tumor tissues (21.9%) were found to be positive for PD-L1. Grade 3 GEPNETs were especially correlated with PD-L1 expression (*p* = 0.008) [22]. Additionally, the researchers found that patients expressing PD-L1 had a shorter progression-free survival time and a shorter overall survival. The presence of PD-L1 was also linked to a higher tumor grade (grade 3) in metastatic GEPNETs. The presence of PD-L1 in these patients could act as a biomarker to predict survival. Ultimately, inhibition of PD-L1 could serve as a novel therapy for patients with metastatic GEPNETs.

Leng et al. reported PD-L1 expression in 13 out of 45 GI neuroendocrine tumor patients. PD-L1 positive tumors were all poorly differentiated NETs [23]. Additionally, there was a significant correlation between the presence of PD-L1 expression and tumor grade. Cives et al. found 22 of their 32 GI NETs positive for PD-L1 expression. Moreover, PD-L1 expression was related to worse outcomes as 5-year overall survival was 32% in PD-L1 positive patients *versus* 90% in PD-L1 negative patients [24].

Tsuruoka et al recently published their results on PD-L1 expression in thoracic neuroendocrine tumors. The authors conducted immunohistochemistry on tissue microarray with E1L3N, a PD-L1 antibody clone. The study samples were examined by two blinded independent investigators. A score of 1 or more was considered to be positive for PD-L1 expression. Out of 227 patients, 46 were typical carcinoids (TC), 6 were atypical carcinoids (AC), 106 were large cell neuroendocrine carcinoma (LCNEC) and 69 were small cell lung carcinoma (SCLC). None of the TC and AC were found to be positive for PD-L1 expression. 5.8% (*n* = 4) SCLC and 10.4% (*n* = 11) LCNEC patients were found PD-L1 positive [27]. It seems the PD-L1 expression is variable among neuroendocrine tumors and high grade neuroendocrine tumors might have higher odds of being PD-L1 positive especially gastroenteropancreatic origin.

There have been two recent reports evaluating prognostic implication of PD-L1 and PD-1 expression in NETs. Fan et al. investigated 80 pulmonary neuroendocrine patients. 51.3% (41) patients had positive PD-1 expression in tumor infiltrating lymphocytes. A multivariate analysis of their study cohort revealed that both PD-1 and PD-L1 expression were independent survival prognostic factors [29]. Kim et al studied prognostic implication of PD-L1 expression in GEPNETs. 24 GEPNET patients were evaluated for PD-L1 expression and authors found that median overall survival in PD-L1 positive patients was 16 months as compared to 24.8 months in PD-L1 negative patients [30].

Table 2 summarizes the ongoing clinical trials evaluating clinical activity of immune checkpoint inhibitors in neuroendocrine tumors.

**Table 2:** Current status of Immune checkpoint inhibitor in prospective clinical trials for neuroendocrine tumors

	NCT ID	Disease cohort	Investigational Agent	Phase	Current Status
Phase 2, Open-label Study of pembrolizumab monotherapy in Patients with Metastatic High Grade Neuroendocrine Tumors	02939651	Stage IV, G3 neuroendocrine tumor	Pembrolizumab 200mg every 3 weeks	2	Recruiting
Study of pembrolizumab With lanreotide Depot for Gastroenteropancreatic Neuroendocrine Tumors (PLANET)	03043664	Stage IV well or moderately differentiated gastroenteropancreatic neuroendocrine tumors (GEP-NETs)	Pembrolizumab 200mg IV every 3 weeks and Lanreotide depot 90mg SQ every 3 weeks	1/2	Recruiting
Study of Efficacy and Safety of PDR001 in Patients with Advanced or Metastatic, Well-differentiated, Non-functional Neuroendocrine Tumors of Pancreatic, Gastrointestinal (GI), or Thoracic Origin or Poorly-differentiated Gastroenteropancreatic Neuroendocrine Carcinoma (GEP-NEC)	02955069	Stage IV, Well differentiated non-functional NET of GI, Pancreas and Lungs Poorly differentiated gastroenteropancreatic neuroendocrine carcinoma	PDR001 dose is 400 mg infusion every 4 weeks PDR001 is a high-affinity, ligand-blocking, humanized IgG4 antibody directed against Programmed Death-1 (PD-1) receptor that blocks the binding of PD-L1 and PD-L2.	2	Recruiting
Pharmaco-immunological Study of Interferon-alpha and Metronomic Cyclophosphamide Association in Neuroendocrine Tumors (EPICentro)	02838342	Grade 1 or 2 metastatic neuroendocrine tumor	Metronomic cyclophosphamide Interferon-alpha	2	Recruiting
Study of JS001 in Patients with Advanced Neuroendocrine Tumors	03167853	Stage IV, Grade 2 or 3, well-differentiated neuroendocrine tumors and poorly-differentiated	JS 001 humanized anti-PD-1 monoclonal antibody	1	Recruiting
Durvalumab (MEDI4736) plus tremelimumab for Advanced Neuroendocrine Neoplasms of Gastroenteropancreatic or Lung Origin (DUNE)	03095274	G1-2 GI, Pancreatic and Bronchial NET and G3 non-thoracic neuroendocrine carcinoma.	Durvalumab, 1500 mg Q4W Tremelimumab 75 mg Q4W	2	Recruiting

## CONCLUSION

Specific clinical trials looking at just GEPNETs and immune checkpoint inhibitors are limited (Table 2) and although the results of these trials are still a few years away, it’s a step in right direction. Neuroendocrine tumors are an area of unmet medical need and despite increasing incidence and prevalence, the treatment options for progressive disease are limited. NET incidence has been rising over the past four decades. We recently presented our 18-year Kentucky Cancer Registry database review at a national neuroendocrine symposium and found multi-fold increase in incidence of NETs from 3.76/100,000 to 10.7/100,000 population [25]. This is consistent with the surge in incidence seen at the national level per SEER database [26]. As most decisions in drug development are affected by pharmacoeconomics, continued efforts from the medical fraternity exploring the role of immune checkpoint inhibitors in NETs might turn the tide.

Our patients certainly benefited from a trial of immune checkpoint inhibitors without any adverse side effects. Overall, we were able to provide some degree of disease stabilization and improved performance status. It is to be noted that all our study patients had otherwise exhausted all standard of care treatment options and were on the verge of hospice. Our single center experience shows encouraging results. We eagerly await results of prospective clinical trials.
